# Neurostimulation and Reach-to-Grasp Function Recovery Following Acquired Brain Injury: Insight From Pre-clinical Rodent Models and Human Applications

**DOI:** 10.3389/fneur.2020.00835

**Published:** 2020-07-21

**Authors:** Charles-Francois V. Latchoumane, Deborah A. Barany, Lohitash Karumbaiah, Tarkeshwar Singh

**Affiliations:** ^1^Department of Animal and Dairy Science, University of Georgia, Athens, GA, United States; ^2^Regenerative Bioscience Center, University of Georgia, Athens, GA, United States; ^3^Department of Kinesiology, University of Georgia, Athens, GA, United States

**Keywords:** reach-and-grasp, stroke, traumatic brain injury, rodent, human, neuromodulation

## Abstract

Reach-to-grasp is an evolutionarily conserved motor function that is adversely impacted following stroke and traumatic brain injury (TBI). Non-invasive brain stimulation (NIBS) methods, such as transcranial magnetic stimulation and transcranial direct current stimulation, are promising tools that could enhance functional recovery of reach-to-grasp post-brain injury. Though the rodent literature provides a causal understanding of post-injury recovery mechanisms, it has had a limited impact on NIBS protocols in human research. The high degree of homology in reach-to-grasp circuitry between humans and rodents further implies that the application of NIBS to brain injury could be better informed by findings from pre-clinical rodent models and neurorehabilitation research. Here, we provide an overview of the advantages and limitations of using rodent models to advance our current understanding of human reach-to-grasp function, cortical circuitry, and reorganization. We propose that a cross-species comparison of reach-to-grasp recovery could provide a mechanistic framework for clinically efficacious NIBS treatments that could elicit better functional outcomes for patients.

## Introduction

Reach-to-grasp is an essential task that requires precise spatial and temporal integration of sensory and motor systems. Damage to these systems following stroke or traumatic brain injury (TBI), commonly leads to long-term deficits in reach-to-grasp function (1, 2). Given that reach-to-grasp movements are a fundamental skill for many daily activities, improving reach-to-grasp recovery after brain injury is a major goal of neurorehabilitation therapies (3, 4). Non-invasive brain stimulation (NIBS) is of interest as both a prognostic tool for predicting motor recovery after brain injury and as a novel option for rehabilitation treatment (5–8). When used as an intervention in clinical populations, NIBS techniques such as transcranial magnetic stimulation (TMS) and transcranial direct current stimulation (tDCS) have been shown to modulate localized regions of activity in the cortex and are administered either independently or in combination with task-specific training to promote functional recovery. Despite growing evidence of the benefits of NIBS in reducing motor impairment after brain injury, there are still large gaps in our understanding of the optimal treatment parameters, the underlying neural mechanisms, and factors that influence outcomes.

Rodent models have the potential to inform how neurostimulation can be useful in clinical applications, especially as it pertains to improving reach-to-grasp behavior after brain injury. The sensorimotor circuitry controlling volitional motor control and related reaching and grasping behavior is highly conserved in mammals, with significant cross-species similarities in posture and usage of forelimbs (9). Rodents exhibit a similar laminar organization of their M1 cortex compared to primates, and proximal and longitudinal connectivity patterns are also known to be mostly conserved (10). In addition to their comparable dexterity and homology to human cortical representation, rodent models offer several translational advantages—such as the availability of transgenic lines, feasibility of invasive function modulation, and imaging tools that can be exploited to study circuit function and behavioral outcomes under highly controlled environments (11, 12).

The purpose of this review is to compare and contrast current literature on reach-to-grasp deficits in pre-clinical rodent models and human studies, and to highlight how neurostimulation in pre-clinical rodent models of brain injury could provide a mechanistic basis for improving reach-to-grasp function in humans. We begin with an overview of the similarities and dissimilarities between the behavioral and neural mechanisms of reach-to-grasp movements in humans and rodents. We then compare how reach-to-grasp circuitry is affected post-injury in humans and rodents and describe the current efficacy of NIBS protocols in clinical applications and rodent models. In each section, we identify future directions and identify potential avenues for clinical translation.

## Comparison of Reach-to-Grasp Function in Humans and Rodents

### Characteristics of Reach-to-Grasp in Healthy Humans

Prehension is the act of reaching and grasping objects during activities of daily living. Picking up a coffee cup, lifting a pen to sign a check, or hammering a nail in the wall—all are movements that involve reaching and grasping an object to achieve a goal. Reaching movements are directed toward extrinsic properties of an object, such as the location and orientation, and involve recruitment of larger arm muscles to transport the hand to the object. Grasping movements are directed toward the intrinsic properties of the object, such as shape and size, and require synchronized recruitment of extrinsic and smaller intrinsic hand muscles to open and close the digits as the hand approaches the object (13). During a typical reach-to-grasp movement, there is first a gradual opening of the grip with straightening of the digits, followed by a progressive closing of the fingers to match the affordance of the object (14). The maximum grip aperture occurs at about 60–70% of the reach trajectory and is strongly correlated with the size of the object (15). Besides object size, the fragility of the object (16), texture, and weight (17) also influence digit pre-shaping during the reach to the object.

**Figure 1:**
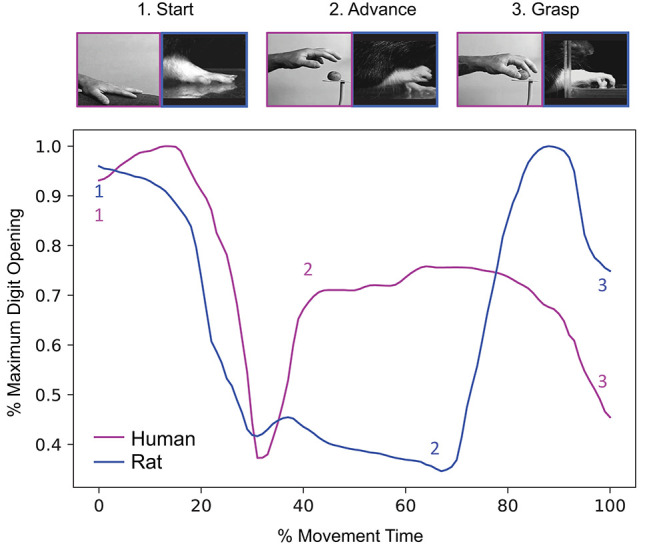
Rodents and humans present high similarity in kinematic stages and coordination. Top panel: Representative hand/paw positions for three stages (start, advance, and grasp). Adapted from (13). Bottom panel: Temporal evolution of maximum digit opening throughout reach-to-grasp, with corresponding timing of each movement stage as a function of overall movement time. While there are differences in kinematic strategies between the human hand and rodent paw, the gross dynamics, and morphology of reach-to-grasp remain the same. Adapted and republished with permission of Elsevier Science & Technology Journals from Sacrey et al. (21); permission conveyed through Copyright Clearance Center, Inc.

Humans typically grasp an object in either a precision or power grasp (18–20). Precision grasps (Figure 1) involve creation of an opposition space between the thumb and the remaining digits and the application of force on the object with digit tips (22). In contrast, power grasps are used when large forces need to be applied to the environment, e.g., while wielding a hammer, and involve the fingers to be flexed in opposition to the palm with the degree of flexion dictated by the dimensions of the object (15, 23).

### Reach-to-Grasp Assessments in Rodents

Much like humans, rodents demonstrate a high level of dexterity and fine usage of the limb while engaging in reach-to-grasp movements. In particular, direct kinematic comparison of reach-to-grasp between rodent and humans shows strong conserved motor and coordination strategies (21, 24) (Figure 1). Skilled reaching tasks in rodents provide multiple measurements that quantify dexterity and ability to perform the task via measures such as the number of hand changes, number of successful hits, efficiency, and response time. The assessment of forelimb function in rodents can be achieved through several modalities that distinguish power and precision grip, as in humans. Power grasp and strength can be simply measured using a Newton meter by allowing the rodent to grasp on a bar while being pulled to evaluate the strength at failure to grab. Assays such as the staircase assay (25), skilled reach task (26), the pasta matrix (27), and the pasta handling assays (28) were designed to assess the skilled use of the forelimb for precision reach and grasp or fine handling of food.

The scoring of hand shape and grasp movement has also been used to evaluate the extent of compensatory changes in movement strategy following injuries (26, 29, 30). More recently, feedback loop approaches have been developed to investigate motor adaptation and motor strength using lever pulling in static (31, 32) and dynamic conditions (33), which allows for a closer investigation of the neural correlates in motor planning and execution during power grasp experiments. Here, our focus is on precision grasp-related assays such as the skilled reach task or reach-and-grasp assay to draw a correspondence with similar assays described in human literature.

### Scalability: Mapping Rodent Experimental Paradigms to Clinical Motor Outcomes

Despite many similarities, there are nonetheless important postural differences in reach-to-grasp movements between rodents and humans. Unlike humans, rodents also have to stabilize overall posture during reach-to-grasp, but this will not be reviewed here as they are exhaustively covered elsewhere (34–36).

A current limitation in using findings from rodent experiments to understand reach-to-grasp impairments in humans is the dichotomy in the scales and outcomes used to score task performance. The rodent literature has mostly focused on pure performance such as successful number of hits, and for the most part, has ignored compensatory mechanisms of task accomplishment, such as altered handshape, unconventional motor strategies, and two-limb assisted performance. In contrast, the human literature has emphasized kinematic and kinetic analyses that isolate specific features of movement that are impaired after injury (37–40). Specific parameters of reach-to-grasp movements are predictive of general upper extremity impairment and function (41). For example, maximum grip aperture is higher in patients following stroke, and is predictive of functional impairments (42). Thus, a closer examination of rodent aperture scaling in response to different haptic-based object cues may provide a window into behavioral measures that are clinically relevant (13).

Overall, pre-clinical animal models would benefit from the incorporation of higher sensitivity assessments in order to distinguish finer motor alterations and associated motor circuitry adaptations following brain injuries (43, 44). Recent advances in low-cost platforms for trajectory tracking (45) and open source software developed for markerless pose estimation (46, 47) makes measuring fine-grained changes in kinematics easily implementable in the lab setting and provides a relevant framework for cross-species comparisons.

## Neural Mechanisms of Reach-to-Grasp

### Neural Mechanisms of Reach-to-Grasp in Humans and Non-human Primates

Reach-to-grasp movements in primates are mediated by two cortical dorsal stream pathways involving cortical interactions between posterior parietal cortex (PPC), premotor cortex (PM), and primary motor cortex (M1) [reviewed in (48, 49)]. Reaching is enabled primarily by a dorsomedial pathway that projects through the superior parietal lobule (SPL) via the parietal reach region, including the superior parieto-occipital cortex, to the dorsal premotor cortex (PMd) and finally to M1 (50–52). The dorsomedial stream uses visual motion information to monitor spatial location of objects to allow for skilled reaching movements to occur (53). In contrast, the dorsolateral pathway is responsible for grasping and projects through the anterior intraparietal sulcus to ventral premotor cortex (PMv) and then to M1. This pathway primarily receives and processes visual information on object affordances (14, 54). The dorsolateral visual stream is also involved in recognition of object motion and self-motion (55). The basal ganglia and cerebellum each form reciprocal connections with the two cortical streams and play an important modulatory role in facilitating eye-hand coordination during corrective online responses and integrating reach and grasp components into a single motor program (56–58).

In addition to these neural substrates, the corticospinal tract also mediates integration of reach-to-grasp movements under visual control (59). Non-human primates and humans have direct connections between corticospinal neurons and the cervical motorneurons. The phylogenetic development of the corticospinal tract also correlates with dexterity, particularly in the ability to perform precision grips (60). Indeed, non-human primates with weaker corticospinal projections have no ability to form precision grips and possess limited manipulatory skills. In humans, TMS-induced motor evoked potentials, a measure of corticospinal excitability, are more suppressed during power grasps than precision grasps, suggesting a more pronounced role of the corticospinal tract in precision grasps (61, 62).

Reaching and grasping movements emerge independently in humans in the early stages of development, and foveal vision plays a critical role in facilitating the integration of these movements during development (13). Young infants primarily rely on proprioception to direct their hands toward objects and use somatosensory feedback to determine if they have contacted an object (63). Between 4 and 8 months of age, infants begin to use vision to organize sequential reach-then-grasp movements (64). By about 9 months of age, infants begin to exhibit feedforward visual control of functional reach movements by orienting the hand to match the orientation of the object. This suggests early ontogenetic maturation of the dorsomedial pathway compared to the dorsolateral pathway. The gradual maturation of the dorsal visual stream during the first decade of life (65) allows smooth integration of reach-to-grasp movements under feedforward visual control (66). In adults, simultaneous processing of visual inputs in these two streams allow the reach and grasp movements to be concurrently executed as a single smooth integrated act (67–70). When visual processing is limited, either during traumatic brain injuries or through experimental manipulation, prehension decomposes into its two elemental components, suggesting disruption in online processing and interactions in these two pathways.

### Neural Mechanisms of Reach-to-Grasp in Rodents

Rodent models have facilitated the detailed investigation of motor circuits using techniques such as regional lesioning, electrical stimulation, and optogenetics. Much like humans, motor circuitry in rodents is also divided into 3 major groups: (1) higher order centers that include posterior parietal (PPC), primary (M1; also identified as the caudal forelimb area—CFA) and secondary/premotor (M2/PM; also identified as the rostral forelimb area—RFA) regions that are involved in sensorimotor integration, planning and volitional control, (2) modulatory and relay centers that include the thalamo-striatal-midbrain axis responsible for gating, selective sensory integration, and motor plasticity, and (3) motor coordination and execution centers such as the cerebello-cortico-spinal pathway that regulate the direct translation of efferent higher order signals into physical movements as well as providing feedback for fine movement tuning and learning. Primary motor regions modulate movement outcomes through both direct and indirect pathways involving corticospinal neurons and reticulo/rubro/tectospinal pathways, respectively (71, 72).

In contrast to the cerebellum and spinal control of posture and locomotion, skilled motor learning is thought to be orchestrated through the corticomotor and motor planning pathways in association with a descending pathway consisting of midbrain and spinal cord neurons (73, 74). In comparison to humans, the corticomotor and planning pathways in rodents are organized through a much reduced PPC mapping to CFA and RFA (75). Cholera toxin B (CTB) retrograde tracing coupled with optogenetic probing of CFA/RFA interconnectivity established asymmetric intra-hemispheric connections between the two regions, with CFA strongly projecting to L2/3 and L5a of RFA, and RFA strongly projecting to L1 and L5b of CFA (76).

**Figure 2:**
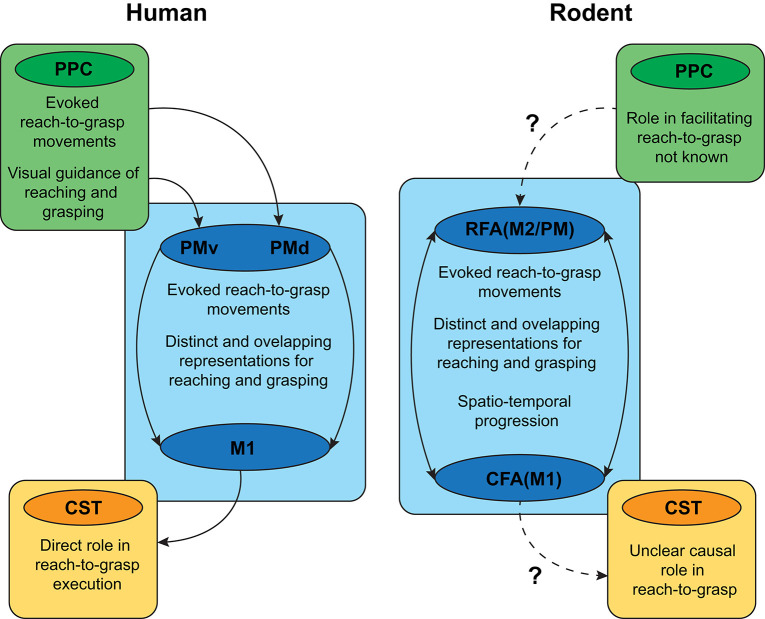
Overview of similarities and differences in the cortical circuitry supporting reach-to-grasp function in humans and rodents. PPC, posterior parietal cortex; CST, corticospinal tract; PMd, dorsal premotor cortex; PMv, ventral premotor cortex, M1, primary motor cortex; CFA/M1, Caudal forebrain area, primary motor cortex; RFA/M2/PM, rostral forebrain area/secondary motor cortex/pre-motor cortex. Solid arrows: known connectivity and circuitry; Dashed arrows: lack of knowledge on connectivity and circuitry.

With regards to reach-to-grasp, there is not a clear distinction of the CFA (primary) and RFA (secondary) function in rodents (Figure 2). Though there is some evidence for preferential reach-related activity in CFA and grasp-related activity in RFA (77, 78), it is believed that CFA and RFA might share the same motor planning and execution function and only differ in how internal state information is integrated with motor planning (71). Recent studies have demonstrated the sensitization of RFA to rewarding situations and internal adaptation to feedback stimuli, which is a key feature of neuroplastic changes and motor control flexibility (71).

The somatotopic organization of the motor cortex mapped using intracortical microstimulation (ICMS) has helped reveal part of the functional association between sub-divisions of the cortico-spinal layer (layer IV-V) of the RFA and CFA (79, 80). Short duration ICMS revealed that isolated body movements of the wrist, elbow, and neck had overlapping representations in RFA and CFA. Long duration stimulations, on the other hand, have been shown to elicit complex motor sequences such as reach, grasp, and retract (81), with distinct representations in RFA and CFA. These results suggest that sustained cortical activation might recruit corticospinal neurons into combined sequences of single movements and might also act as a basic template for the learning of skilled motor tasks. In summary, although rodents share homologous neural circuits with humans, the functional segregation of CFA and RFA in the rodent cortex is less distinct when compared to M1 and PM in the human cortex, suggesting that targeting anatomically analogous regions need not necessarily elicit similar functional outcomes.

### Scalability: Defining the Overlap Between Rodent and Human Cortical Circuitry for Reach-to-Grasp

In addition to a shared somatotopic organization related to different parts of the body, motor cortical regions may contain spatially distinct representations of complex movements, such as reach-to-grasp, of the animals' natural motor repertoire (82, 83). The similarity between ethological “action maps” across species lends support to the view that a common organizational structure supports reach-to-grasp movements in humans, non-human primates, and rodents (81, 84, 85). The differential extent of cortical mappings devoted to a given movement category, between species (and across individuals within-species) depends on the behavioral relevance and specific experience with performing that movement type (86, 87). This suggests that rodent models of reach-to-grasp can focus on functional, rather than strictly anatomical, correspondence across interacting motor cortical areas to best inform mechanisms of human reach-to-grasp.

Rodent models can also improve our understanding of the functional specialization related to the neural substrates of reach-to-grasp action. In primates, though reaching and grasping components have been localized to dorsomedial and dorsolateral pathways, respectively, recent neurophysiological and neuroimaging evidence has revealed overlapping reach- and grasp-related neural activity in each pathway (88–91). Likewise, rodent models have associated CFA with the reaching/retracting motions associated with more proximal forelimb muscles and RFA with grasping movements associated with distal forelimb muscles (77, 78), whereas others have shown each area plays a role in both reaching and grasping (92, 93). This has led to the view that the posited functional segregation between reaching and grasping may simply be a byproduct of overlapping representations that are activated sequentially during naturalistic reach-to-grasp movements (94). Consequently, translating rodent models of reach-to-grasp to humans will depend on a stronger characterization of how the spatiotemporal progression of neural activity in rodent CFA and RFA maps onto a similar progression in human cortical pathways.

The main disadvantage of rodent models concerns evolutionary development of specialized neural circuits for more sophisticated control of prehension in primates (95). Though both rodents and primates have many descending motor cortex projections through the CST, lesions to the CST have a stronger effect on reach-to-grasp movements in primates than in rodents (96). The presence of direct monosynaptic connections between a phylogenetically newer portion of M1 and cervical motoneurons in primates make CST a prominent contributor to reach-to-grasp movements (59, 97, 98). The diverging function of CST in rodents and primates mirrors the behavioral advantage of primates for fractionated digit control and visual guidance for precision grip (13).

Relative to the motor cortex, fewer investigations of reach-to-grasp have targeted rodent PPC. Though PPC in rodents serves roles across a wide variety of sensory, cognitive, and motor domains (99, 100), whether rodent PPC contains complex movement representations similar to primates is unclear (101). Anatomically, the relative size of PPC in rodents suggests a much smaller role in coordination of reach-to-grasp movements. Functionally, a recent investigation by Soma et al. (102) suggests that PPC's contribution to the control of movement in rodents fundamentally differs from that of primate PPC: whereas neurons in primate PPC show a preference for movements of the contralateral limb (as with M1 and PM), neural activity in rat PPC showed a strong ipsilateral preference.

In summary, rodent models of neural organization show the greatest promise in elucidating the underlying spatial structure and temporal dynamics of premotor and motor areas throughout the course of a reach-to-grasp action. However, they have limited scalability in terms of understanding the role of associative regions and descending pathways in reaching and grasping function.

## Loss Of Reach-to-Grasp Function Following Brain Injury

### Reach-to-Grasp Dysfunction Following Brain Injury in Humans

Impaired reach-to-grasp after stroke is most commonly observed as a result of upper limb paresis following damage involving the CST or cortical motor areas (103–105). After stroke, paresis mainly affects the side of the body opposite the lesion, in addition to subtler deficits observed ipsilesionally (39). Stroke survivors with damage to the CST generally exhibit slower arm transport velocities, decreased movement smoothness, and more segmented hand opening during grasp (39, 42, 106), while other features, such as the temporal synchrony between the transport and grasp phases, are relatively preserved (42, 106, 107). In TBI, diffuse brain damage can lead to arm paresis and other motor impairments (108). Motor function is relatively preserved relative to neurocognitive effects, this may be partly because the standard clinical measurements do not capture the full extent of sensorimotor deficits after TBI (109, 110). Children with moderate or severe TBI showed slowing of both trajectory and grip formation in a reach-to-grasp task, and differences in performance persists months after TBI onset (111). Other observed deficits in movement preparation and execution are similar to those seen post-stroke, though recovery of function is likely to be different given the large disparities in post-injury plasticity (1, 108).

Behavioral changes following brain injury are coupled with substantial reorganization of cortical networks, and the extent of cortical reorganization beyond the focal tissue damage can have large facilitative or maladaptive effects on motor function and recovery (112, 113). Spontaneous reorganization mainly occurs within the first 3 months post-stroke in humans, a critical time period in which heightened plasticity can be exploited to facilitate recovery (114). The recent advances in brain connectivity analyses have led to a greater emphasis on understanding remote effects of the infarct on large-scale brain network structure (115–119). A meta-analysis found that across a wide variety of motor tasks and impairments, stroke patients exhibit higher activation in primary motor cortex opposite the lesioned hemisphere, as well as increased bilateral premotor activity relative to healthy controls (120). During performance of simple movements, TBI patients show altered brain activation patterns and connectivity within the motor network (121, 122). Though less is known about connectivity with parietal regions, recent neuroimaging studies suggest that weakened connections between the parietal, premotor, and motor areas underlie motor impairments after stroke (123, 124). In both TBI and stroke, changes in parietofrontal networks can persist even in patients who are well-recovered, suggesting a long-term reorganization of functional networks subserving compensatory motor planning and execution (108, 125).

Much of the focus in understanding cortical reorganization has concerned an altered balance of excitatory and inhibitory inputs (E/I balance). TMS has been used extensively as an assessment tool to probe the excitability of motor cortex and functional CST integrity via measurement of motor-evoked potentials. In early stroke, patients without upper-limb motor evoked potentials exhibited considerably worse functional recovery than patients with observable motor evoked potentials (126). When used in combination with standard clinical assessments of upper-limb impairment and MRI-based biomarkers of CST integrity, TMS-based assessments of corticospinal excitability strongly predicted potential (or lack of potential) for motor recovery after stroke (127, 128). More severe CST damage, especially damage to fibers originating in primary motor cortex, is associated with impaired hand function and poorer rehabilitation outcomes (129–131). TMS GABA receptor function and tonic GABA concentration, markers of inhibition, are generally reduced in the affected hemisphere post-stroke (132–134). In severe TBI, reduced corticospinal excitability is related to both greater diffuse axonal injury and greater motor impairment (135, 136).

Changes in relative excitation and inhibition and their relevance for motor recovery after ischemic stroke depend on the severity and location of the lesion. Di Pino et al. (137) proposed a “bimodal-balance recovery model,” in which the optimal interhemispheric interactions between motor cortices depends on the available neural pathways post-injury. According to the model, in patients with relatively low impairment (and preserved neural pathways), increased inhibition of hemisphere opposite of the lesion onto the lesioned hemisphere is maladaptive, whereas in patients with severe impairment (and more damaged neural pathways), an interhemispheric imbalance constitutes compensatory plasticity supportive of recovery, perhaps a consequence of shifting toward more ipsilateral control via the cortico-reticulo-propriospinal pathway (138). Likewise, the role of premotor cortex depends on impairment severity—whereas in less impaired patients, ipsilesional PM plays a larger role through increased connectivity with M1 and descending corticospinal tracts, in more impaired patients contralesional PM facilitates M1 to aid in movement execution (139, 140). However, recent evidence suggests that interhemispheric imbalance only emerges after the sensitive period when changes in motor behavior are minimal (141, 142). Thus, how targeting restoration of E/I balance may facilitate recovery post-injury remains an open question.

### Motor Cortex Injury and Reach-to-Grasp Function Loss in Rodents

Stroke and TBI in rodent models cause sizeable lesions and significant cell death in brain tissue. Despite similar manifestations of hemorrhage and edema across both stroke and TBI, their pathophysiologies are quite different. Stroke can be ischemic (75% of all cases), hemorrhagic (15% of all cases) or both (143), leading to reduced blood perfusion of the adjacent brain tissue that causes an immediate loss of neuronal activity and functional impairment. The state of prolonged ischemia (oxygen depletion) triggers massive cell death that induces progressively larger lesions due to secondary insults mediated by excitotoxic agent release (neurotransmitters) and neuroimmune responses. Severe TBI results from a blunt force injury or severe penetrating lesions to brain tissue that leads to shearing of neuronal circuits and vasculature. The progressive damage of brain tissue in the days, months, and years thereafter are a result of the secondary injury cascades that are similar to stroke as described above. Following a severe TBI, the massive loss in tissue integrity in the lesion site and surrounding regions is due to the significant cell death and breach of the blood-brain barrier (BBB), which results in permanent loss of function.

The functional impairments observed in rats following TBI have been shown to be dependent on age (144), lesion size, and depth (145), as well as the presence of an edema (146). The extent of tissue loss dictates the initial functional impairment and the speed of recovery. Rodents subjected to small focal impact injuries (<3.0 diameter) might recover up to 90% of their initial pre-injury performance within 15 days (145). However, the same extent of recovery is not evidenced in the case of larger and deeper lesions, especially those spanning the pyramidal tract and white matter, with animals never recovering more than 80% of initial performance within 4 weeks post-TBI (146). The intervals between testing post-injury vary considerably across studies, which makes discriminating between causative factors of recovery quite challenging.

Severe TBI studies in rodents have demonstrated that the motor cortex undergoes reorganization post-TBI, involving either intra-cortical inhibition (147–150), inter-hemispheric crosstalk, axonal sprouting (151), or cholinergic/dopaminergic-dependent plasticity disruptions (152). Similar to humans (see *Reach-to-Grasp Dysfunction Following Brain Injury in Humans section*), an increase in activity in perilesional tissue and complementary motor regions such as PM, is indicative of the reorganization of motor function following injury (153–155). Motor reorganization and compensatory mechanisms observed in rodents are often associated with a return of function to near pre-lesion performance levels (146). It is now well-established that the extent of cortical reorganization and its associated re-functionalization, or lack thereof, is heavily dependent on the extent of exposure to stimulating environments and tasks prone to induce neuroplastic changes such as enriched environments and novel motor tasks (152, 156, 157), which also form the basis of some rehabilitative strategies used in humans (155, 158, 159).

Direct investigations of circuit refunctionalization after TBI using voltage-sensor dye imaging of the entire cortex showed a long-lasting, delayed, and decreased response of the ipsi- and contralateral cortex following forelimb stimulation (sensory). Whereas, direct optogenetic stimulation of the RFA cortical surface revealed a strong asymmetric contralateral response indicating the presence of a spontaneous inter-hemispheric rewiring at week 8 post-injury (160). These results supported the notion that motor learning of the intact limb (contralesional cortex) is further enhanced and might provide compensatory support for task completion (161). Indeed, these findings are further supported by observations indicating expansion of the contralesional somatotopic motor map following TBI, and studies demonstrating intact limb restraint leading to limited recovery of the impacted limb (162). In addition, restraining of the intact limb limited recovery potential of the impacted limb (146). Altogether, these results suggest that plasticity changes and motor reorganization of the hemisphere contralateral to the lesion might partially support functional recovery after TBI in rodents.

The convergence and overlap of peri-lesional, intra-cortical, inter-hemispheric, and systemic compensations promote a spontaneous reorganization and offloading of motor function following injury. All these plastic changes might ultimately be driven by trophic factor-dependent mechanisms such as those activated by brain-derived neurotrophic factor (BDNF) (163–166), which facilitates spontaneous rewiring-driven functional recovery observed in rodents after exercise rehabilitation, and potentially after drug and neurostimulation driven repair.

### Scalability: Translating Rodent Injury Models to Post-injury Reorganization in Humans

Cortical reorganization of upper-limb function using ICMS protocols in rodents, and TMS/MRI in humans, have been instrumental in detecting enhanced perilesional and contralesional neural activity and excitatory/inhibitory (E/I) imbalance, both acutely and chronically post-injury. The E/I imbalance has been shown to drive acute cortical depression that further enhanced secondary excitotoxic cascades that accelerated intra- and perilesional neuronal death (167). It is therefore important to note that E/I imbalance might also be targeted to shape cortical reorganization post-injury through the promotion of long-term potentiation and depression (168–170). Dialysis and magnetic resonance spectroscopy studies in humans found E/I imbalance similar to that observed in rodents (171), however, the direct correspondence between rodent and human responses to a loss of E/I homeostasis remains to be explored.

Despite profound disparities in the anatomical correspondence in the CST between rodents and humans (see *Scalability: Defining the Overlap Between Rodent and Human Cortical Circuitry for Reach-to-Grasp section*), it is now evident that the extent of CST loss post-injury might proportionally correspond to the observed contralesional hemispheric activation (146). The induction of controlled cortical impact lesions of brain tissue and quantification of refunctionalizaton post-injury is quite feasible in rodent studies when compared to humans, where comorbidities and lesion variabilities might provide a very restricted spatial and mechanistic understanding of injury progression.

The reach-to-grasp task is considered a skilled learned task that involves the acquisition of specific and fine-tuned sequences of movements (172). As such, it has been demonstrated that skilled motor learning as opposed to other forms of repetitious motor learning tasks induces a reshaping of the motor cortical landscape (173) that is dependent on intra-cortical integration of sensory and planning information (174, 175), and on the cholinergic (152, 176) and the dopaminergic (177, 178) afferent projections to the motor cortex. Importantly, the morphology, laminar distribution and density of cholinergic basal forebrain innervation varies greatly between humans and non-human primates, let alone between humans and rodents (179, 180). Although the laminar distribution of cholinergic systems and control of plasticity by cholinergic inputs is highly conserved between rodent and humans (181, 182), the specific role of this system in motor learning and post-injury adaptation remains to be elucidated.

Despite several disparities, the shared principles of reorganization between rodent and human models such as anatomical and physiological changes, E/I imbalance, regional compensatory activity, and impact of environmental factors, point to the translational relevance of pre-clinical rodent studies.

## Neuromodulation for Motor Recovery Post-Brain Injury

### Neurostimulation to Promote Motor Recovery: Clinical Studies

The goal of non-invasive brain stimulation (NIBS) therapies is to augment existing rehabilitation protocols through shaping the cascade of reorganizational processes that occur post-injury. NIBS protocols aim to either upregulate preserved areas of cortex supporting effective recovery or downregulate areas that are thought to be maladaptive. Two of the most common methods in human clinical trials are repetitive transcranial magnetic stimulation (rTMS) and transcranial direct current stimulation (tDCS). In each of these methods, the after-effects of stimulation remain for minutes or hours beyond the stimulation itself, allowing for behavioral testing of effects or pairing stimulation with other rehabilitation interventions. rTMS involves a train of magnetic pulses for a set duration, intensity, interval, and frequency. Low-frequency (≤1 Hz) rTMS delivered continuously for 10–30 min induces inhibition of cortical excitability, whereas high-frequency (5–20 Hz) increases cortical excitability. A more recent form of rTMS is theta-burst stimulation (TBS), in which patterned pulses are delivered in triplets at 50 Hz either continuously (every 200 ms for 20 or 40 s) to increase cortical excitability (continuous TBS; cTBS) or intermittently in 10 bursts repeated every 10 s for 192 s to decrease cortical excitability (intermittent TBS, iTBS). TBS is appealing in clinical settings due to its shorter duration, lower intensity, and longer lasting after-effects relative to LF- or HF-rTMS (183).

In tDCS, application of low-intensity (1–2 mA) currents through two electrodes placed on the scalp for 5–30 min increases or reduces excitability in a polarity-specific manner. When compared to rTMS, tDCS has worse spatiotemporal resolution but generally has longer after-effects. Although more recently, high-definition tDCS has been used to improve spatial resolution (184, 185). Though TMS and tDCS have different mechanisms of action, they are both thought to activate mechanisms of neuroplasticity that are similar to long-term potentiation or long-term depression (186), both at the stimulation location and remotely (185). Typically, intervention studies investigating NIBS and motor recovery have designed stimulation paradigms based on principles of the interhemispheric inhibition model, either by exciting ipsilesional cortex (HF-rTMS, iTBS, anodal tDCS), inhibiting contralesional cortex (LF-rTMS, cTBS, cathodal tDCS), or both (187, 188).

**Figure 3:**
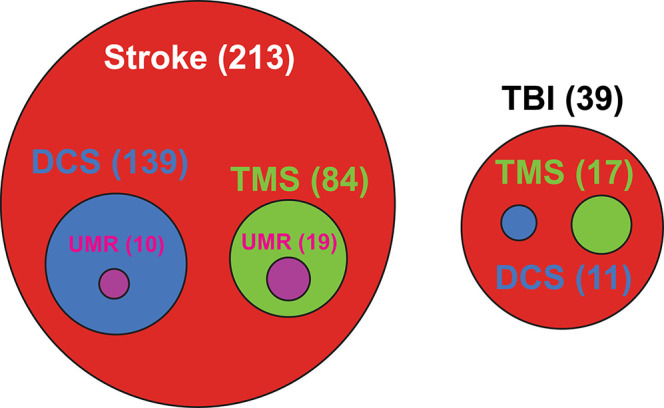
Current landscape for clinical trials investigating non-invasive brain stimulation treatments for stroke and traumatic brain injury. TBI, Traumatic brain injury; DCS, Direct current stimulation; TMS, Transcranial magnetic stimulation; UMR, Upper-limb motor rehabilitation. Number in parenthesis represents the number of matching clinical trials related to NIBS treatment. Source: https://www.globalclinicaltrialsdata.com/; data post-filtered for matching key words on conditions and treatments.

There is currently a greater emphasis on implementing NIBS protocols for stroke than for TBI recovery. Of the currently registered clinical trials using rTMS or tDCS for treatment of stroke or TBI, 85% are focused on stroke recovery (as of Dec 2019—Figure 3). Research on the applicability of NIBS as an intervention for recovery of motor deficits is in the nascent stages in TBI as compared to stroke (6, 189, 190). Despite this rapidly increasing interest in clinical applications, there is little consensus on the efficacy of such treatment protocols [for systematic reviews and meta-analyses, see (191–198)]. The mixed effectiveness across studies is a confluence of many interrelated factors. In healthy humans, factors such as demographics, genetic polymorphisms, anatomical features, and current brain state all contribute to response variability (183). Compounding these differences, in patients, the level of initial functional impairment (mild to severe) and associated functional integrity of the CST (e.g., presence or absence of TMS-induced motor-evoked potentials) further influences which NIBS treatment protocol (excitation or inhibition) is most likely to be effective (199). Response to treatment also depends on the timing relative to the injury—whereas the goal of stimulation in the acute and subacute phases may be to augment motor learning during spontaneous recovery, in the chronic phase NIBS may instead be geared toward “re-opening” the sensitive period and/or focusing on compensation. From an experimental design perspective, the specific parameters selected, including intensity, duration, focality of stimulation, and number of stimulation sessions, can influence the directionality of changes to cortical excitability and treatment efficacy (200–202). Finally, how NIBS is paired with behavior modulates effectiveness, with treatment effects often most promising when stimulation is applied during motor training (185, 203, 204).

Few NIBS applications thus far have specifically pinpointed kinematic-based reach-to-grasp outcomes after brain injury, though many studies have used clinical assessments of dexterity (e.g., Jebsen-Taylor Hand Function Test, Perdue Pegboard Test) or tests with reaching and grasping subcomponents (e.g., Wolf Motor Function Test, Action Research Arm Test) (191, 205). Nowak et al. (206) examined the effects of LF-rTMS to the contralesional M1 in a cohort of patients with subcortical strokes with mild to moderate motor impairment. Consistent with the bimodal-balance recovery model, they found that reducing contralesional M1 excitability in this cohort resulted in improved peak wrist velocities and peak digit apertures of the affected hand, matching kinematic performance of their unaffected hand. Similarly, LF-rTMS to the contralesional hemisphere improved peak aperture and a composite measure of reach-to-grasp coordination, especially for grasps to smaller objects (207, 208). LF-rTMS and TBS have also been shown to augment effects of reach-to-grasp and precision grip training in chronic stroke patients (209, 210). Finally, Lefebvre et al. (211) found improved precision grip kinematics following bilateral tDCS, in which anodal stimulation over ipsilesional M1 and cathodal stimulation over contralesional M1 was applied simultaneously. Collectively, these studies provide evidence that the positive effects of NIBS on motor function can extend to more complex manual dexterity.

Despite obvious benefits, whether NIBS can aid in the recovery of complex motor functions like reach-to-grasp remains an open question. Clinical NIBS studies to date have almost exclusively targeted M1 for both practical (e.g., determining optimal stimulation coordinates) and theoretical (interhemispheric competition models) reasons. Compared to improvements in simple motor parameters, stimulating M1 sometimes have a muted effect on complex motor functions (212). Thus, given the wealth of evidence of widespread changes to cortico-cortical interactions governing motor control after brain injury, there is a need to investigate whether other nodes in the parietofrontal system are suitable stimulation targets for improving reach-to-grasp behavior. Supporting this notion, Lotze et al. (213) found that stimulating contralesional M1, PMd, and SPL each led to deficits in performance of a complex sequential motor task in well-recovered stroke patients, suggesting that all three areas play a role in compensatory reorganization. Though PMd has recently received more attention as a potential stimulation target for improving dexterity following brain injury (214, 215), similar investigations targeting PPC in reach-to-grasp function are lacking.

### Neurostimulation to Promote Motor Recovery: Pre-clinical Models

Current clinical strategies to treat post brain injury trauma following a stroke or a TBI are mostly designed to alleviate immediate symptoms such as intracranial pressure, blood clots, overall blood perfusion and control of hemorrhage (216, 217). Despite the enormous physical, medical, and economic burden associated with post-brain injury, to this date, no clinical treatment is available for the repair and re-functionalization of the damaged brain (218–220).

NIBS techniques such as rTMS and tDCS/DCS allow for a high temporal and spatial resolution control of neuronal ensemble activity in humans as well as in rodents. The pre-clinical investigation of neurostimulation and reach-and-grasp has substantially improved our understanding of the underlying mechanisms of recovery post-injury, facilitated by combining NIBS with more invasive approaches. Mechanistically, in the rodents, TMS and DCS modulate excitability of cortical structures (221–223), BDNF-dependent motor learning (224–226), neurogenesis (227, 228), and can result in improved motor performance (229). Overall, the use of NIBS in rodents can be broadly classified into two distinct approaches:

#### Schedule Stimulation and Skilled Motor Recovery

It has been shown that the severity of the motor cortex injury, and particularly, the extent of damage to corticospinal neurons can seriously impair spontaneous recovery in rats (146). M1 electrical stimulation targeting the contralesional cortical column, including corticospinal neurons, promoted corticospinal axonal sprouting and re-functionalization at the contralesional spinal cord tracks, leading to improvements in reaching (230–232). While these previous studies used intensive and long stimulation protocols, more acute application of tDCS has also been shown to facilitate functional motor recovery (233, 234). Similarly, monopolar fast DCS could improve skilled reaching in rats over a 10 day period (235, 236). Such scheduled stimulation might cause transient responses in activated microglia (237, 238).

#### Pairing Neurostimulation and Behavior for Targeted Causal Recovery

**Figure 4:**
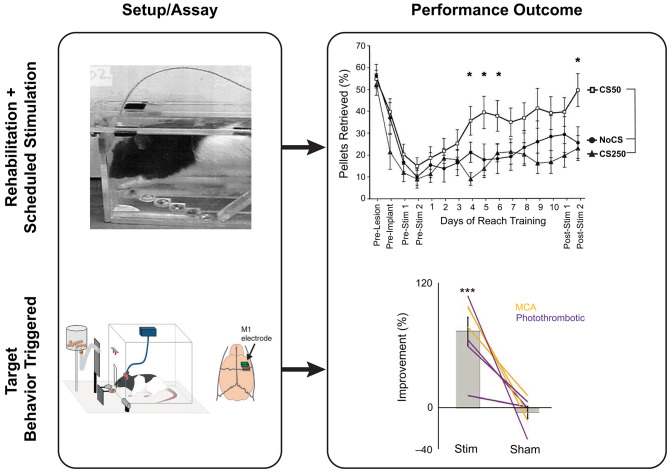
Neurostimulation strategies and reach-to-grasp recovery in rats post-injury showing two typical stimulation paradigms. Top panel: Scheduled rehabilitative stimulation using the staircase task on rodents post-controlled cortical impact injury and tDCS at various frequencies of stimulation. Bottom panel: Behavior-triggered stimulation. Using an automated skilled reach task box, the timing of stimulation can be performed relatively to the intended movement to reinforce cortical plasticity and recovery post-stroke in rats. Adapted from Ramanathan et al. (153), reprinted with permission from Springer Nature, Nature Medicine (advance online publication, 07/2020, doi: https://doi.org/10.1038/s41591-018-0058-y), and Adkins-Muir and Jones (233), reprinted by permission of Taylor & Francis (Taylor & Francis Ltd, http://www.tandfonline.com). *p < 0.05 and ***p < 0.001.

Ramanathan et al. (153) have shown in rodents that the RFA (rostral forelimb area; M2/PM) produces short, slow oscillatory local field potentials for the duration of the reach-grasp-retract phase of a single pellet retrieval assay which disappear, intra- and peri-lesionally, following stroke along with a reduction in retrieval performance. Interestingly, the authors paralleled a similar loss in sensorimotor synchronization in patients with stroke performing a center-out reaching task. Timely triggering of DCS stimulations could re-ignite peri-lesional slow oscillations and reach-to-grasp performance. The cross-functionality of the RFA and CFA was demonstrated in another study where a CFA injury resulting in loss of reach-to-grasp performance could be recovered through closed-loop DCS re-pairing of the primary sensory cortex (S1) and RFA, resulting in a functional stream that bypasses the CFA and facilitates faster reach-and-grasp recovery (239). Pairing neurostimulation with behavior has a major advantage of creating associations with the stimulation in a reliable manner, leading to faster recovery (Figure 4). Ultimately, it is expected that neurostimulation paired with behavioral tasks would have a longer-lasting re-functionalizing impact than neurostimulation alone.

Altogether, these results show that rodent models allow for a wide exploration of parameters and combinatorial approaches, while providing mechanistic evidence for their efficacy.

### Scalability: Using Pre-clinical Models to Refine Neurostimulation Parameters for Clinical Use

Pre-clinical use of neurostimulation has been essential for optimizing the wide parameter space that encompasses NIBS, as well as to define the essential mechanistic components that direct positive outcomes following stimulation such as, neurogenesis, neural stem cell proliferation/migration (240, 241), the triggering of plasticity enhancing pathways (224), the promotion of excitability (242, 243), dendritic spine and axonal sprouting (244) and the direct pairing between volition and reach-and-grasp control (153, 239). Yet, the role and translational relevance of these factors in mediating recovery in humans after stroke or TBI is not entirely clear and remains to be investigated.

**Figure 5:**
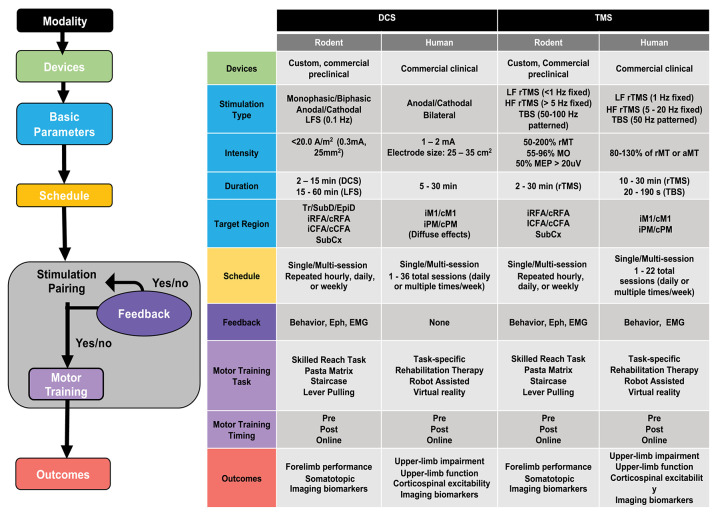
Decision tree for designing rehabilitative paradigm using non-invasive brain stimulation (NIBS). Left panel: Flow chart illustrating the various steps for designing a NIBS-based rehabilitative paradigm. Right panel: Table summarizing common, non-exhaustive ranges of parameters and protocols used for direct current stimulation (DCS) and transcranial magnetic stimulation (TMS) techniques in rodent models and human studies of motor recovery following acquired brain injury. LF, low frequency; HF, High frequency; TBS, theta burst stimulation; LFS, Low frequency stimulation; rTMS, repetitive TMS; rMT, resting motor threshold; aMT, active motor threshold; MO, maximum output (TMS device); MEP, motor-evoked potential; Tr, transcranial; SubD, subdural; EpiD, Epidural; SubCx, subcortical; i/c, ipsi/contralateral; RFA, rostral forelimb area; CFA, caudal forelimb area; M1, motor cortex; PM, premotor cortex; CST, corticospinal tract; Eph, Electrophysiology; EMG, electromyography. Representative parameters drawn from (153, 191, 194, 196, 198, 224, 233, 239, 249, 250).

Clinical studies remain very segregated from pre-clinical findings, and promising results from pre-clinical studies remain largely untested in humans (245). The disconnect between applied research and clinical practice suffers in part from medical conservatism and “guesswork” around known stimulation parameters. This divide could be bridged to a significant extent by evidence from pre-clinical research studies, which offer the exploration of stimulation parameters (246) and that could help reduce guesswork and improve chances of success in human testing (247). We propose that the exploratory data obtained from rodent studies could be integrated in human clinical trials by replacing current clinical protocols by relevant pre-clinically defined parameters within a safe and applicable framework (248). This information can be integrated into a decision-tree approach to select cross-cutting or distinct parameters, and behavioral pairing combinations, which can lead to favorable functional outcomes (Figure 5).

Several studies have explored the effectiveness of tDCS stimulation in rats post-TBI. The comparison of tDCS parameters such as polarity (251) and train stimulation frequency (233), in combination with rehabilitation (252, 253) or intact limb-restriction (254), have demonstrated that the cathodic train stimulation of 100 Hz paired with motor rehabilitation could be used as an optimal tDCS-based rehabilitation scheme for enabling reach-to-grasp function recovery post-TBI—although this remains to be tested in humans. More recently, Ramanathan et al. (153) identified the initiation of slow oscillation pre-reach-to-grasp movement in rats and humans, and their causal relationship to upper-limb performance. Using a behavior-triggered stimulation regimen that reproduced the observed slow oscillations in the peri-lesional motor area, they demonstrated that paired behavior-stimulation could enhance reach-to-grasp performance. These examples illustrate stimulation parameters and timing in pre-clinical rodent studies, that have promising translational potential in humans. More importantly, these preclinical results suggest that NIBS parameters that target cortical plasticity, rather than E/I imbalance, have significant benefits, especially when performed in combination with upper-limb rehabilitation.

There are translational alternatives for when DCS parameters are not directly applicable to humans, particularly for closed-loop and repeated train stimulation parameters, which encompass rapid and abrupt changes of current. For example, transcranial alternative current (tACS) offers a less irritable and sudden change in stimulation than tDCS, which might be more suitable for human applications, as reflected in a growing body of literature showing effectiveness in both rodents and humans to treat brain injury (255, 256). In addition, online rTMS approaches that time stimulation onset to changes in movement, muscle activity, or brain state are novel avenues to approximate closed-loop stimulation in rodent models (257, 258).

Key challenges and questions need to be addressed to ease translation between rodent models and human studies of NIBS to improve motor recovery after brain injury (Table 1). For example, both rodent and human studies would benefit from incorporating a shared set of kinematic assessments in order to better quantify and translate motor outcomes (259). This can not only help resolve open questions about cortical organization for reach-to-grasp action (e.g., what is the functional correspondence between rodent CFA/RFA and human M1/PM?), but would provide a common framework for comparing stimulation parameters across species (e.g., does stimulation parameter A in rodents lead to a similar change in reach-to-grasp kinematics as stimulation parameter B in humans?). Rodent models could benefit from a more deliberate focus on stimulation devices and protocols that can be feasibly and safely implemented in humans, and to incorporate systematic heterogeneity in the study population as a way to address the high variability in individual responses to NIBS interventions in clinical trials (246, 260). Likewise, clinical studies can continue to optimize strategies for pairing NIBS with motor behavior to improve functional recovery (204). Finally, both studies in rodents and humans could explore novel potential targets beyond M1, both to inform our understanding of network plasticity mediating reach-to-grasp actions following brain injury and to potentially tailor interventions to individuals.

**Table 1:** Cross-cutting recommendations and open questions related to pre-clinical and clinical assessments of reach-to-grasp function.

	**Recommendations**	**Open questions**
Outcome measures	Include standardized measurement of RTG kinematics	What are the main similarities/differences in RTG kinematics in rodent models and patient populations?
Study population	Asses similar study protocols for RTG recovery in TBI and strokeIn clinical studies, incorporate biomarkers to stratify based on factors such as CST integrity, lesion size/location, and genetics	How does responsiveness to NIBS differ in TBI vs. stroke? What mechanisms can account for the large intra- and inter-individual variability in response to NIBS?
Stimulation parameters	In rodent models, focus on parameters and protocols within human safety guidelines Establish a correspondence between rodent and human NIBS parameters based on comparable motor performance	What are most promising candidates for stimulation types/patterns to test in humans? What are similarities/differences in dose-response relationships in rodents and humans?
Target region	Evaluate predictors of responsiveness to NIBS of ipsilesional/contralesional M1 and PM Test PPC as a candidate target for RTG recovery	How do M1/PM/CST differentially contribute to RTG performance in rodents and humans? What regions or protocols in rodents show similar RTG outcomes to stimulating PPC in humans?
Motor training	Focus on protocols that combine NIBS with concurrent motor training Explore timing stimulation to movement kinematics	Does task-specific training with NIBS generalize to overall improvements in motor function? Can NIBS selectively target separable components of RTG function to improve recovery?

*RTG, reach-to-grasp; TBI, traumatic brain injury; NIBS, Non-invasive brain stimulation; CST, Corticospinal tract; M1, primary motor cortex; PM, premotor cortex; PPC, Posterior parietal cortex*.

## Conclusion

In this review, we present the idea that the reach-to-grasp movement is an impaired motor function following acquired brain injury, such as traumatic brain injury or stroke. Though important dissimilarities in neural architecture and the behavioral response to injuries exist in humans and rodents, overall the reach-to-grasp movement presents a strongly conserved neural circuitry across the two species. Similar responses to brain injury and to treatment post-injury demonstrate that the rodent model could serve as a testbed for translational rehabilitation of brain injury using non-invasive brain stimulation (NIBS). We propose that pre-clinical parameters should be considered in current clinical trials to improve functional outcomes. Currently, clinical trials using NIBS are primarily focused on stroke, but TBI might deserve as much interest as stroke due to its prevalence, and in turn, because it is a major risk factor for stroke (261). More importantly, for both stroke and TBI, the rehabilitative applications and clinical trials targeting upper-limb recovery and reach-to-grasp are still largely lacking. The considerable knowledge associated with volitional motor control of reach-to-grasp should be used as a steppingstone to deepen our understanding of functional remapping post-injury as well as functional recovery post-NIBS.

## Author Contributions

C-FL and DB edited the data and figures. C-FL and LK analyzed the data base entries. C-FL, DB, TS, and LK wrote and edited the manuscript. All authors contributed to the article and approved the submitted version.

## Conflict of Interest

The authors declare that the research was conducted in the absence of any commercial or financial relationships that could be construed as a potential conflict of interest.
